# Cross-Depicted Historical Motif Categorization and Retrieval with Deep Learning

**DOI:** 10.3390/jimaging6070071

**Published:** 2020-07-15

**Authors:** Vinaychandran Pondenkandath, Michele Alberti, Nicole Eichenberger, Rolf Ingold, Marcus Liwicki

**Affiliations:** 1 Document, Image and Video Analysis Group (DIVA), University of Fribourg, 1700 Fribourg, Switzerland; michele.alberti@unifr.ch (M.A.); rolf.ingold@unifr.ch (R.I.); 2 Staatsbibliothek zu Berlin—Preußischer Kulturbesitz, 10785 Berlin, Germany; nicole.eichenberger@sbb.spk-berlin.de; 3 EISLAB Machine Learning, Luleå University of Technology, 97187 Luleå, Sweden; marcus.liwicki@ltu.se

**Keywords:** historical document, watermarks, image retrieval, convolutional neural network, deep learning

## Abstract

In this paper, we tackle the problem of categorizing and identifying cross-depicted historical motifs using recent deep learning techniques, with aim of developing a content-based image retrieval system. As cross-depiction, we understand the problem that the same object can be represented (depicted) in various ways. The objects of interest in this research are watermarks, which are crucial for dating manuscripts. For watermarks, cross-depiction arises due to two reasons: (i) there are many similar representations of the same motif, and (ii) there are several ways of capturing the watermarks, i.e., as the watermarks are not visible on a scan or photograph, the watermarks are typically retrieved via hand tracing, rubbing, or special photographic techniques. This leads to different representations of the same (or similar) objects, making it hard for pattern recognition methods to recognize the watermarks. While this is a simple problem for human experts, computer vision techniques have problems generalizing from the various depiction possibilities. In this paper, we present a study where we use deep neural networks for categorization of watermarks with varying levels of detail. The macro-averaged F1-score on an imbalanced 12 category classification task is 88.3%, the multi-labelling performance (Jaccard Index) on a 622 label task is 79.5%. To analyze the usefulness of an image-based system for assisting humanities scholars in cataloguing manuscripts, we also measure the performance of similarity matching on expert-crafted test sets of varying sizes (50 and 1000 watermark samples). A significant outcome is that all relevant results belonging to the same super-class are found by our system (Mean Average Precision of 100%), despite the cross-depicted nature of the motifs. This result has not been achieved in the literature so far.

## 1. Introduction

The identification and retrieval of historical watermarks has been an important research field for codicology and paper history for a long time [1]. The main use of watermark identification is dating of historical papers, for example when cataloguing non-dated medieval manuscripts [2]. There are also broader research questions addressed by watermark identification [3,4], e.g., economical history research. Watermarks are created during the process of handmade paper-making from tissue rags, as was done in Europe from the Middle Ages (13th century) till the mid-19th century [1]. The paper making process involved plunging a mould into liquid tissue pulp. On the metal mesh-work of the mould, the paper makers fixed a small figurine made of wire which produced the watermark on the paper. These moulds were typically used for around two years and then replaced. This makes it possible to use watermarks for dating [2], i.e., if two paper sheets have the same watermark, we can assume that they were produced within the same two year period. If one of them is dated, we can infer the origin date of the other with an accuracy of plus or minus two years.

### 1.1. Problem Statement

**Figure 1:**
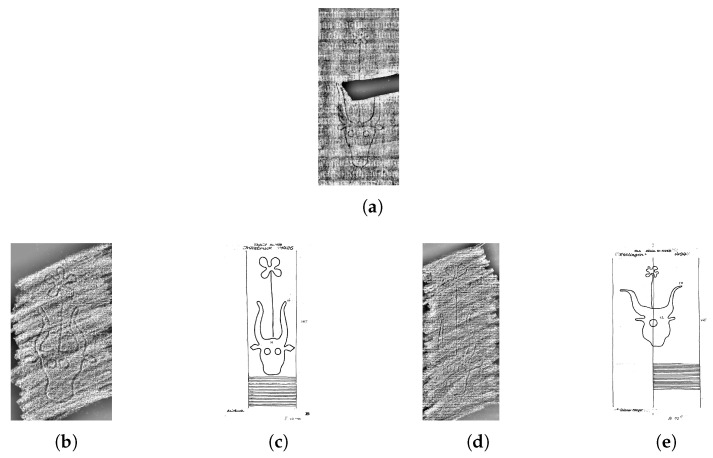
Example of a query (**a**) with the expert annotated results in order (**b**–**e**) where (**b**) is the most relevant. Our system retrieves these results among the first six ranks in the order (**e**,**d**,**b**,**c**). Please note that the system is not affected by the different reproduction techniques (a: radiography, b/d: rubbing, c/e: tracing).

A fundamental problem of watermark reproductions in databases is the diversity of acquisition methods (e.g., hand tracing, rubbing, thermography, digital photography, and radiography). This leads to depictions of the exact same watermark in radically different ways (see Figure 1). While being of utmost importance for watermarks, it is a common problem for handwritten historical documents in general, where other elements (letters, motifs, and decorations) could also be depicted in several different ways [5,6,7,8]. This problem is known as the cross-depiction problem [9], which refers to the challenges faced by computer vision methods when identifying an object, even when it is depicted in different visual forms.

Content-based image retrieval methods were used to tackle automatic image retrieval since the early 1990s [10]. Image retrieval tasks face two general problems: the intention gap, which refers to the difficulty of formulating a precise query based on the visual information needs of users, and the semantic gap, which means that users often search for visual information with abstract concepts, but automatic image retrieval mainly works on concrete properties, often based on raw pixels. This leads to the problem that two objects can represent the same concept, but be very different at a visual level, and also, two objects can be very similar on the visual level but belong to completely different concepts [11].

Previous approaches to automatic watermark identification show that watermarks are a topic that has been addressed quite early by image retrieval research, but these approaches lack the precision—in terms of correct class and other fine-grained detail-like size of watermark, exact distance between the chainlines and the postition of watermark in relation to the chainlines—necessary to facilitate complete replacement of manual retrieval processes by historians.

Besides in our own previous work [12], the possibility of applying deep learning on cross-depicted watermarks (or motifs in general) has not been investigated so far. This is also due to the fact that neural networks in general often fail at identifying the abstract concept and can therefore easily be fooled by noise [13] or abstract depictions [14]. In this paper we show that this general statement does not apply to cross-depicted watermarks, especially when a good training strategy and loss function is used.

### 1.2. Contribution

The main contribution of this paper is an in-depth study of deep learning approaches on classifying and retrieving cross-depicted historical watermarks. With the final goal of developing a content based image retrieval system for cross-depicted historical watermarks, we first approach the task of classification (macro-averaged F1-score of 88.3% on an imbalanced 12-class problem), followed by fine-grained multi-labelling performance (Jaccard Index [15] of 79.5% on 622 labels). We then formulate the problem as one of image similarity matching using the weights of the models trained in the previous two tasks (mean Average Precision score of 0.95). Finally, we assess the performance on expert-defined similarity-ranking ground-truth for a small dataset (50 items, 5 motif classes) and a large dataset (1000 items, 10 motif classes). The retrieval rate of our system is perfect (100% Mean Average Precision) with only the ranking of the retrieved relevant results differing from the expert’s opinion.

Please note that we reported early indications that deep learning-based approaches can tackle the cross-depiction problem in [12]. This current article, however, is a completely new study, where we (i) introduce the dataset and discuss the problem of the existing classification systems in detail, (ii) investigate multiple advanced deep learning architectures to tackle the problem, (iii) perform novel experiments on a more fine-grained multi-labelling task (622 labels), (iv) design a similarity matching system which perfectly retrieves relevant motifs out of a new 50-item dataset and 91.4% of the relevant symbols out of a 1000-item dataset (created and validated by a domain expert).

## 2. Related Work

In this section we summarize the most relevant related work for our case.

### 2.1. Cross-Depiction

A lot of work has been done in the context of object classification, object detection and image similarity but very few of them specifically address the problem of depicting an object in different ways. The most relevant work in our context is Hall et al. [9] who raised awareness of the difficulty of the problem and how both traditional methods and deep learning methods fail to solve it. Recent work that used deep learning include Shen et al. [16] who used spatially consistent feature learning to identify near-duplicate works in art collections and Fei et al. [17] who used a deep visual semantic descriptor fusing low and high level features for sketch-based image retrieval.

Other works mainly employ hand-crafted methods, including Crowley and Zisserman [18] who attempted object retrieval in paintings, Hu and Collomosse [19] used HOG descriptor for sketch-based image retrieval, Rakthanmanon et al. [20] introduced a motif discovery algorithm that allows for detecting similar sub-images across documents using the Generalized Hough Transform. En et al. [21] proposed a local descriptor-based algorithm for spotting patterns in historical documents and Ginosar et al. [22] used four methods for detecting People in Cubist Art using a deformable part model.

### 2.2. Automatic Image Retrieval Applied to Watermarks

Automatic image retrieval has been identified as an interesting technology for historical document research quite early. Watermark retrieval has been addressed as a use case for Content-Based Image Retrival (CBIR) methods in the late 1990s by Rauber et al. [23] and Belov et al. [24]. In the Northumbria watermarks archive project [25] a CBIR system originally developed for trademark retrieval has been applied to tracings and radiographies of historical watermarks. The system worked much better on tracings than on radiographies [26]. However, there were no experiments conducted on datasets with mixed reproductions.

Brunner [27] also conducted experiments on a homogeneous dataset of around 1800 watermarks (all scanned with the same method) belonging to 14 different motif classes with a CBIR system using local and global features. He observed that the performance of the retrieval system varied significantly depending on the motif classes. According to Brunner, two problems lead to the poor performance: high visual variance within one class and overlap between motifs of different classes. He also attempted classification using a Support Vector Machine, but only achieved a true positive rate of 87.41%.

Otal and van der Lubbe [28] address the problem of isolation and identification of identical watermarks. While they achieve a result of 91% correctly identified watermarks, it should be considered that their test-case involved a simple task with only 170 watermarks and one motif class. Additionally, all of their watermark reproductions were created with a uniform method.

Picard et al. [29] propose a retrieval system for watermark classification that works with local features and a dictionary trained on a large amount of data. Their tests are performed on a homogeneous set of 658 watermarks tracings. These watermarks are divided into groups labelled as relevant results for query images, but it remains unclear how many motif classes are used and how the relevant results are determined. The evaluation measure is the rank of the first relevant result which would be at 168 in a random ranking. In the tests they achieve a average rank of the first relevant result between 30.6 and 61.7 depending on the parameters used. This however, is not particularly helpful for watermark researchers who would have to look at 30–60 results for each query to find a relevant hit.

Another project on image retrieval for watermarks is currently being conducted at the Ecole nationale des chartes, the Institut National de Recherche en Informatique et en Automatique (INRIA) and the Institut de Recherche et d‘Histoire des Textes (IRHT) [30]. In this project, they aim to identify only identical watermarks, with each class consisting of multiple images (in the same or in different reproduction techniques) of the a single watermark. Opposed to this, our work aims at finding similar (not only identical) watermarks. While it is interesting to identify the exact watermark for the precise dating of manuscripts, it is also important to be able to retrieve the class membership of a watermark in the broader classification system and to find similar watermarks as this can facilitate the work of scholars in the humanities. The retrieval of similar watermarks is particularly helpful in scenarios where the scholars are not very familiar with a particular classification system, or when there exists no identical watermark to a given query.

There are many commercial and non-commercial CBIR systems available and there have been several initiatives to test and evaluate different systems [31,32], but due to the velocity of technical developments in this area, these lists often become rapidly outdated. For our experiments, we chose the IOSB image retrieval system and LIRE because these systems are non-commercial, still being maintained, and equipped with a Graphical User Interface (GUI) that allows for a simple query by image process. The last point was important because in our interdisciplinary work, some of the experiments were conducted by a Humanities scholar. These systems are described in the following sections.

#### 2.2.1. IOSB Image Retrieval System

The IOSB image retrieval system was developed between 2010 and 2013 as part of the European research project “FAST and efficient international disaster victim IDentification” (FASTID) at the Fraunhofer-Institut für Optronik, Systemtechnik und Bildauswertung (IOSB) in Karlsruhe with the goal of identifying missing persons and disaster victims using images of their tattoos [33]. Tattoos are usually classified in motif classes, but there is a broad variation of motifs inside the classes and there are overlaps between the classes. In that regard, the task is similar to the classification of historical watermarks. The IOSB system works with local features such as the Scale Invariant Feature Transforms (SIFT) which proved to be more effective at tattoo retrieval than global features. However, there was no optimization of the software for the specific case of watermark retrieval.

#### 2.2.2. LIRE Image Retrieval System

The Lucene Image REtrival (LIRE) system is a Java-based open source application first released in 2006 [34]. It is based on the search engine Lucene and it uses global as well as local features. The global features are low level features concerning color and texture and some combined features. The results presented in this paper were obtained with the freely available demo version of LIRE, using only global features (Fuzzy Color, Texture Histogram and MPEG7 color layout).

### 2.3. Deep Learning

The developments of modern deep learning techniques have led to remarkable improvements in the field of computer vision. When the ImageNet [35] challenge for object recognition was released in 2009, the top ranks were dominated by traditional heuristic-based methods. Only few years later, the well-known model AlexNet [36] opened the road to what would be a cascade of deep learning models which would perform increasingly better every year. In fact, soon afterward the first deep neural networks surpassed human-level performance [37] and yet these results continue to be outperformed by the latest models [38].

Despite these outstanding results, we know that neural networks do not perform vision in the same way as humans. There are many situations in which this difference is very clear. For example, one can look at the diversity in the inherent nature of adversarial examples for humans and computers. While humans can be fooled by simple optical illusions [39], they would never be fooled by synthetic adversarial images, which are extremely effective at deceiving neural networks [13,40]. Another way to analyse the difference is to look into what types of error humans and networks are more susceptible to when performing object recognition.

Deep learning models tend to make mistakes with abstract representations whereas humans are very robust towards this type of error [14]. Successfully identifying an abstract representation—such as drawings, paintings, plush toys, statues, signs or silhouettes—is a very simple and very common task for humans, yet machines still struggle to cope with it.

## 3. Dataset

To identify similar or identical watermarks, many printed collections of watermarks were assembled [1,41,42], and during the last two decades, several online databases were created [1,43,44]. One of the most popular databases—especially for medieval paper from the Middle and Western Europe—is the Wasserzeichen-Informationssystem (WZIS) (https://www.wasserzeichen-online.de/wzis/struktur.php) [44,45,46]. In this work we use a dataset derived from the WZIS database. Our dataset contains around 105,000 watermark reproductions stored as RGB images of size 1500×720 pixels (Not all images have the same size. The numbers reported are the average over the whole dataset). The different image characteristics between tracings (pen strokes, black and white) and the other reproduction methods (less distinct shapes, grayscale) makes the task of watermark classification and recognition more difficult (see Figure 1).

Our dataset contains mainly hand tracings (around 90%), and the remaining images are rubbings and radiography images. In the WZIS database, other reproduction techniques such as thermography images and digital photographies can be found, which are not included in our dataset but have similar characteristics as rubbings or radiography (grayscale, noisy background).

### 3.1. Source of the Cross-Depiction: Data Acquisition

In contrast with handwriting and other types of historical documents, the standard digitization process is not sufficient to get a useful reproduction of a watermark. In fact, the watermarks are invisible to humans under normal conditions. Therefore, special techniques need to be used and this greatly influences the degree of automation achievable in the acquisition of watermark reproductions.

**Figure 2:**
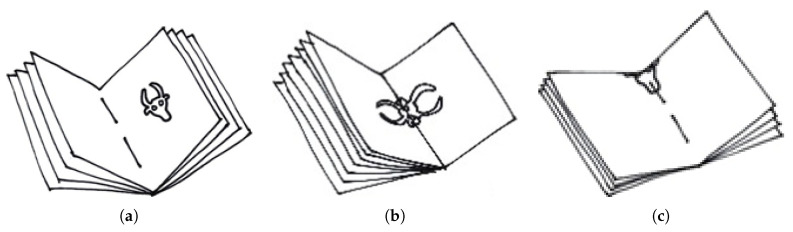
An example of positioning of a watermark on the page. It was common practice to produce big sheets of paper and fold them in order to achieve the desired page size. The amount of folds directly influences the possible locations of the watermarks on the final product. The location of the watermark with three different possible folds are shown in the diagram—one fold in (**a**), two folds in (**b**) and three folds in (**c**).

To localize the watermark, the page is held against a light. Depending on the format of the manuscript, the watermark may be fragmented due to the folding of the paper to obtain the quires (see Figure 2). This significantly increases the difficulty of recognizing it, and in certain cases makes it even impossible.

For automatic watermark retrieval and identification, the reproduction techniques are very important because of their different visual features. Several reproduction techniques were established, such as tracing (see Figure 1c), rubbing (see Figure 1b), radiography (see Figure 1a) and thermography [1,47,48]. Tracing is the simplest and cheapest, but also the least precise technique since it is subject to human interpretation. In the tracing process, the watermark and surrounding chainlines are traced by hand with a pencil on a sheet of tracing paper. Out of the mechanical reproduction techniques, rubbing is the simplest technique, where tracing paper is placed above the watermark and gently hatched (shaded) using a soft pencil. However, it does not work with all kinds of paper and it can be hampered by fragmented watermarks and tight bindings in smaller manuscripts, such as those shown in Figure 2b,c. The more sophisticated techniques such as radiography and thermography [48] serve better results at the expense of being more expensive and time-consuming. Thermography reproductions are digital photographs produced by a thermographic (infrared) camera, and radiographic reproductions require the usage of X-rays to create a negative image of the watermark on an underlying film. Nowadays radiography is no longer used because of its elevated costs and health risks associated with the procedure [1,24,47].

Other techniques include multi-spectral and digital photography, but unlike radiography, they are sensitive to the source text which is sampled along with the watermark [47]. Therefore, in datasets built from medieval books, the most common techniques are still rubbing and hand tracing.

### 3.2. Classification System

In watermark research, there exist very complex classification systems for the motifs depicted by the watermarks. For watermark researchers, the classification system plays a major role for both the manual retrieval and the label-assignment of a watermark. The user must be able to determine the singular correct class for a given watermark. This is not always easy because of ordering issues arising from the classification systems used in the humanities (see Section 3.2.1).

**Figure 3:**
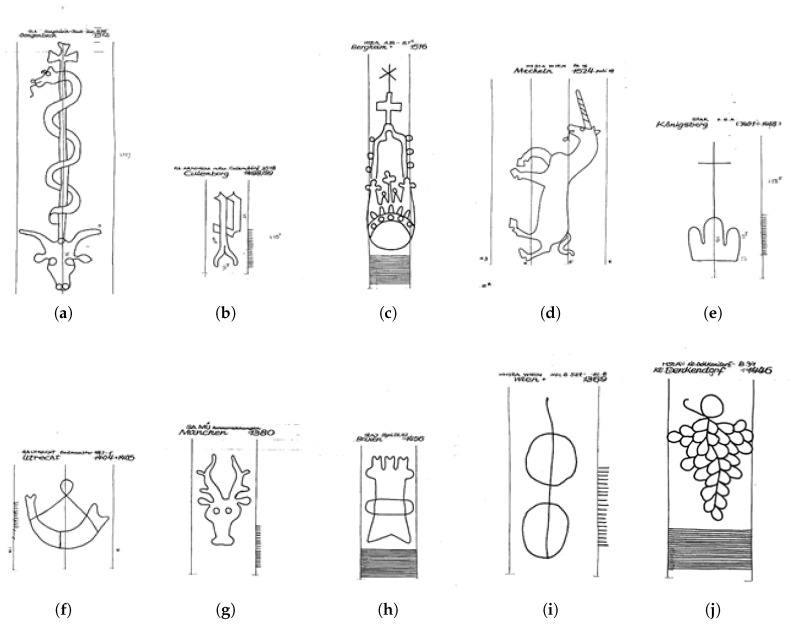
Representation of the class motifs for all 10 classes of TestSet1000. A motif class contains watermarks with similar motifs, but their shape and/or size can vary considerably. Thus, sacrificing precision in the definition for the sake of intuition, one can interpret it as the average class representative. (**a**) Bull’s Head; (**b**) Letter P; (**c**) Crown; (**d**) Unicorn; (**e**) Triple Mount; (**f**) Horn; (**g**) Stag; (**h**) Tower; (**i**) Circle; (**j**) Grape.

Over the years, very complex and differentiated watermark classification systems were developed [43,45,49]. For this work we use the WZIS classification system as it is a widespread standard in the considered domain [45]. It is partially based on the Bernstein classification system [43] and it is built in a mono-hierarchical (In practice, this means that regardless of the level of depth for class specification, there can be only one unique parent class) structure [49,50]. It contains 12 super-classes (Anthropomorphic figures, Fauna, Fabulous Creature, Flora, Mountains/Luminaries, Artefacts, Symbols/Insignia, Geometrical figures, Coat of Arms, Marks, Letters/Digits, Undefined marks) with around 5 to 20 sub-classes each [45]. The super-classes are purposely abstract and are only useful as entry point for classifying an instance of a watermark. For example, the following hierarchy applies for the watermark represented in Figure 3a:

Fauna
  Bull’s head
    detached, with sign above
      with rod consisting of two lines
        serpent (snake) (upright)
          on rod with latin cross
            circle
              enclosed
                with eyes and nostrils

The actual definition is complete only at the ninth level. This kind of terminology is not trivial to be dealt with. Moreover, the user needs special knowledge not only about the single terms but also about their usage in the different scenarios (see Section 3.2.1 for details).

#### 3.2.1. Limitations of the Classification System

**Figure 4:**
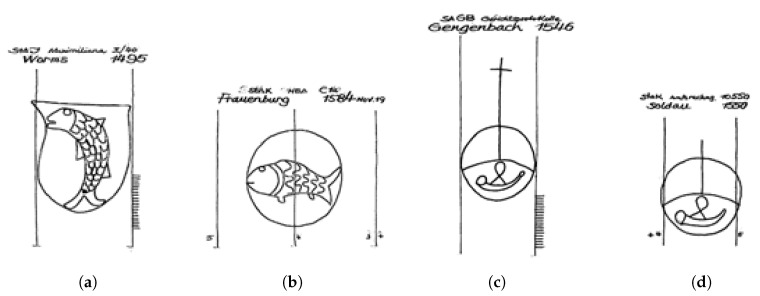
Two examples of an issue caused by the mono-hierarchical tree structure used for classification of the different watermarks (see Section 3.2). Note how sample (**a**) is classified in the superclass “Coat of Arms” and sample (**b**) is classified in the superclass “Fauna” despite looking very similar. This effect can also be observed between samples (**c**,**d**) which despite their similarity, get assigned to two different super-classes. (**a**) Fish on coat of arms. Assigned to coat of arms superclass; (**b**) Fish in a circle. Assigned to fauna superclass; (**c**) Horn in a circle with a cross. Assigned to artefacts superclass; (**d**) Horn in circle. Assigned to geometrical figures superclass.

The mono-hierarchical tree structure mentioned in Section 3.2 often leads to classification problems due to its inflexibility. As the hierarchy forces discriminative decisions in fixed positions, it causes an ordering issue, and that might not be desirable especially from an historical point of view. Such decisions could be acceptable for practical purposes, but lead to issues as the one depicted in Figure 4.

From a computer science point of view, this problem could be interpreted as strong noise in the class labels. However, the pitfall of the classification system is deeper and it greatly affects users who want to retrieve all images which look alike. For example, let’s say that we are interested all watermarks with a fish on it. Since there is the hierarchy “Fauna → Fish”, we would expect to find all relevant images under “Fish”. However, this is not the case. One of the super classes in the WZIS classification system is “Coat of arms”. It includes all images with a coat of arms on them. The catch is that in these “Coat of arms” watermarks there could be several independent motifs, which are also stand alone classes somewhere in the hierarchy, such as “Fish”. Thus, Figure 4a is classified as “Coat of arms → Common charges → Fauna → Fish” which is not where we initially expected to find it.

Unfortunately, the assumption that the frame of the image (e.g., the coat of arms) is the entry point in the hierarchy does not hold, as the different entry points are not consistent. Indeed, one counter example is Figure 4b where there is a fish-motif which is inscribed in a circle. This is classified as “Fauna → Fish → in Circle” instead of the expected “Geometrical Figure → Circle → Fauna → Fish”. It is then counter intuitive that the motif fish is once accessed as content (e.g., in fauna) and once per container (e.g., in coat of arms). To deal with this issue the users need to have a lot of experience with the classification system.

Additionally, it can be that similar watermarks end up in totally different places. While Figure 4c is classified as “Artefacts → Music instruments → Horn → In circle → With additional motif → Cross → Consisting in one line”, Figure 4d is classified as “Geometrical figure → One element → Circle → With additional motif → Horn → Circle with transverse and longitudinal line”. The two figures not only look similar, but they also share a similar size (29 mm and 30 mm) and time stamp (1546 and 1550). Please note that because of the difficulties of reproducing watermarks from manuscripts it cannot be excluded that the cross is not really missing but it has not been captured in the reproduction phase, or that the watermark figurine has deteriorated during the paper making process and the cross part broke away. To make this kind of consideration a user would need to be able to retrieve all images which are similar to a given one, but this is hard because of the classification system. A solution to this problem might be a faceted classification [49], which takes into account all motifs parts of a watermark on an equal level. This could be achieved by modelling the classification schema in more flexible data models such as ontologies.

In addition to the problems induced by the hierarchical structure, there is another problem related to the terminology being used. To have a standard classification system it is first necessary to reach an agreement in terms of terminology to employ. Such an agreement does not exist yet and is difficult to reach because of linguistic and domain specific traditions in the humanities.

On the other hand, even if such an agreement were to be found, a user would then need a significant amount of experience with the terminology and the classification system to navigate it with ease and efficiency. For this reason, there are icons in the WZIS dataset representing the class motifs which help the user and provide basic orientation through the data. In this context, content based image retrieval is a solution which could deal with this serious problem in the field.

### 3.3. Test Sets Description

In this work we present the results obtained on two expert-defined test sets: “TestSet50” and “TestSet1000”, where the number in their name denotes their size. TestSet1000 is composed of images sampled from 10 sub-classes: bull’s head, letter P, crown, unicorn, triple mount, horn, stag, tower, circle, and grape. Examples of these motif classes can be seen in Figure 3. TestSet50 is composed of images sampled from 5 sub-classes: bull’s head, letter P, crown, unicorn, and grape. The choice of these classes is either motivated by how often they appear in the database (e.g., bull’s head, letter P), or by their complexity (e.g., grape, triple mount). The reproduction techniques in these test sets are mixed (hand tracing, rubbing, radiography). It is important to note here that these test sets contain watermarks that are not used in any of the splits used in the three experimental formulations explained in Section 4.

Annotating a dataset for image retrieval is challenging due to the nature of the annotations; for each query in the dataset, it is necessary to rank the images in the dataset in order of their relevance to the query. This makes it more expensive to annotate a dataset for retrieval as compared to classification.

Additionally, it requires a very high level of expertise with both the watermarks and the classification system to produce such queries. Considering these factors, we have a relatively large set of queries for our data. For TestSet50 we have 50 expert-annotated queries—one for each image in the set. For the TestSet1000 we have 10 expert-annotated queries—one for each motif class in the set. In a practical usage scenario, automatic classification of watermarks with regard to their motif classes can be a first step to facilitate entry into the complex classification system. However, the desideratum would be not only to automatically assign the correct class, but also to retrieve the best results on a more precise level. In watermark research, it is usual to assign different relevance/similarity levels to the retrieved samples with regard to the query watermark, such as “identical” (which means originating from the same watermark mould at about the same time), “variant” (which means originating from the same mould but with slight differences due to the mechanical deterioration of the mould); “type” (which means the motifs are the same, but other features such as divergent size prove that the watermarks do not originate from the same mould); “motif group” (which means that the watermarks have the same motifs but their shape and/or size are considerably different), and “class” (which designates motif similarity on a more abstract level).

The relevance level is crucial for the precision of the dating. “Identical” and “variant” samples can usually be dated with a precision of around 5 years, whereas for “type” and “motif group” the dating range must be extended to 10–20 years or more, depending on the retrieved samples. To assess the precision of the similarity ranking, we created Ground Truth at 5 relevance levels (with 4 the highest and 0 the lowest) for the TestSet50: Identical/variant = 4, type = 3, motif group = 2, class = 1, and irrelevant = 0.

## 4. Experimental Setting

Our goal is to develop a content-based image retrieval system for historical watermarks that can help researchers in the humanities. The challenges are numerous and include the cross depiction problem (see Section 3 and Section 3.1) and the lack of a robust classification system (see Section 3.2.1). In this work, we want to leverage the additional information provided by the hierarchical structure present in the WZIS dataset in order to improve retrieval performance. Due to the lack of consistency and flexibility in the labelling system, we try different approaches to train a deep learning system that produces useful embeddings for later use in an image retrieval setting. We formulate the problem in three different ways explained hereafter and perform all experiments using the DeepDIVA experimental framework [51].

### 4.1. Architecture

Deep neural networks are known to be difficult to train because of multiple reasons. One of the major issues is the gradient vanishing problem. In recent years, different solutions were proposed to address this issue, e.g., Long Short-Term Memory (LSTM) [52] networks and Residual Networks (ResNet) [53]. The former is a type of recurrent neural network that is best suited to dealing with sequential data (e.g., timeseries) and is not the most suitable to work with images. The latter is a variant of a Convolutional Neural Network (CNN) which uses skip connections to prevent the gradient from vanishing even in extremely deep networks. This approach was further developed and combined with dense connections (DenseNet) which connects each layer to every other layer in a feed-forward fashion [54]. These two architectural paradigms—namely skip-connections for ResNet and dense-connections for DenseNet—are the state-of-the-art for computer vision tasks. In our work we chose to run our experiments with two different networks, one for each paradigm. Specifically, we use the vanilla implementation of ResNet-18 (∼11.5 million parameters) and DenseNet-121 (∼8 million parameters) from the PyTorch vision package (https://github.com/pytorch/vision/).

### 4.2. Classification

The first approach is to treat the problem as a object classification task. This task is well established in the field of computer vision and it involves assigning a given image with a single class label appropriate to the object in the image [55]. Typically, each image contains only a single object and is thus assigned a single label. The WZIS watermark dataset uses a complex hierarchical classification system that exposes 12 super-classes as entry points (see Section 3.2). We discard the rest of the hierarchical structure and select only these 12 super-classes as labels for the watermarks and train our network (see Section 4.1) to minimize the cross-entropy loss function shown below:

(1) $$L\left(\vec{x},y\right)=-log\left(\frac{e^{{\vec{x}}_{y}}}{\sum_{i=0}^{|\vec{x}|}e^{{\vec{x}}_{i}}}\right)$$

where *x* is a vector of size 12 representing the output of the network and y={0.11} a scalar, representing the label for a given watermark.

#### 4.2.1. Setup

We train two deep CNNs, namely the ResNet-18 and the DenseNet-121 as described in Section 4.1 with changes in the final layers. Instead of using randomly initialized versions of these models, we use variants that were trained for the classification task on the ImageNet dataset from the ImageNet Large Scale Visual Recognition Challenge [56] (ILSVRC). Studer et al. [57] and Singh et al. [58] have previously demonstrated that ImageNet pre-training can be beneficial for several other domains including document analysis tasks.

All input images are pre-processed by removing the whitespace around the image in an automated manner, and then resized to a standard input size of 224 × 224. The dataset is then split into subsets of size 63,626 for training, 7082 for validation and 34,825 for testing. During training, we scale the loss proportionately to the inverse of the frequency of the class to prevent the network from overfitting to the imbalanced distribution of data across the classes. The weight updates for the network are performed using the Stochastic Gradient Descent optimizer with a learning rate of 0.001, momentum 0.9 for 10 epochs.

#### 4.2.2. Results

We evaluate the performance of the systems using the macro-averaged F1-score metric which accounts for imbalanced classes in single-label classification tasks. From Table 1, we can see that the DenseNet outperforms the ResNet by a small margin on all subsets of the dataset (as defined in Section 4.2.1).

### 4.3. Multi-Label Tagging

The second approach is to treat the problem as a multi-label tagging task. Differently from regular classification, in this case each image can be assigned one or more corresponding labels. In our case, this approach is motivated by hierarchical structure of the labels, which, although very noisy, could provide additional entropy thus increasing the performance of the network. It must be noted that—as pointed out in Section 3.2.1—the lower levels of the hierarchy might be inconsistent and not correct from an historical point of view. We, however, treat each level of the hierarchy as a “tag” thus addressing the problem of having one label appearing at different levels in the hierarchy (e.g., the fish class motifs as mentioned above). We train our network to minimize the binary cross-entropy loss function shown below:

(2) $$L\left(\vec{x},\vec{y}\right)=-\sum_{i=0}^{n}{\vec{y}}_{i}\cdot log\left({\vec{x}}_{i}\right)+\left(1-{\vec{y}}_{i}\right)\cdot log\left(1-{\vec{x}}_{i}\right)$$

where *n* is the number of different labels (or “tags”) being used, *x* is a vector of size *n* representing the output of the network and *y* is a multi-hot vector of size *n*, i.e., it is a vector of values that are 1 when the corresponding tag is present in the image and 0 when it is not.

#### 4.3.1. Setup

The setup for this task is similar to Section 4.2.1 with a few changes. To prepare the dataset for multi-label tagging, first we determine the number of unique labels (3064) (see Section 3.2 for examples) in the dataset and then eliminate all labels which do not have a support of at-least 100 samples in the dataset, leaving 622 labels in the dataset. Please note that we still use the splits defined in Section 4.2.1, and only the labels are changed. Additionally, due to the complexity of the task, we train the network for a longer duration of 50 epochs with an initial learning rate of 0.001 decayed by a factor of 10 every 20 epochs.

#### 4.3.2. Results

We evaluate the performance of the systems using the Jaccard Index [15] which is also referred to as the Mean Intersection over Union (IoU). This metric is used to compare the similarity of sets which makes it suitable for multi-label classification problems. In Table 2, we see that both networks achieve a high degree of performance on the tagging task, with DenseNet performing significantly better on all subsets of the dataset (as defined in Section 4.2.1).

This result is quite significant as the IoU accounts for imbalance in the labels. That is, for a dataset with *n* classes, even if a class is significantly over-represented in the dataset, it contributes only 1/n towards the final score. Please note that *n* equals 622 in this case.

### 4.4. Image Similarity

Lastly, we treat the problem as image similarity task, i.e., given an image produce an embedding in a high dimensional space where similar images are embedded closer and dissimilar images are embedded far apart. This is intuitively the closest formulation to our end goal of image retrieval. There are different approaches to formulate this task in the literature, such as two-channel networks [59], advanced application of the Siamese approach such as MatchNet [60] or the triplet loss approach [61]. We chose to adopt the triplet loss approach which was shown to outperform the other approaches in several benchmarks [61,62]. The triplet loss operates on a tuple of three watermarks {a,p,n} where *a* is the anchor (reference watermark), *p* is the positive sample (a watermarks from the same class) and *n* is the negative sample (a watermarks from another class). The neural network is then trained to minimize the loss function defined as:

(3) $$L\left(\delta_{+},\delta_{-}\right)=max\left(\delta_{+}-\delta_{-}+\mu ,0\right)$$

where $\delta_{+}$ and $\delta_{-}$ are the Euclidean distance between anchor-positive and anchor-negative pairs in the feature space and μ is the margin used.

#### 4.4.1. Setup

The setup for this task is similar to Section 4.2.1 with a few changes. First, we use the same network architectures as defined in the previous tasks modifying only the final layer. The final classification/tagging layer is ablated and replaced with a 128-dimensional fully connected layer. This is done such that the network outputs an embedding of input images in a high-dimensional space where similar images are closer (by measure of Euclidean distance) to each other than dissimilar images.

Secondly, instead of only comparing the two different network architectures, we use the models pre-trained for the Classification task (see Section 4.2 and the Multi-Label Tagging task (see Section 4.3). We hypothesize that the networks that were pre-trained for the other two tasks may have learned weights/filters specific to the WZIS dataset. These new filters or weights might result in increased performance on the similarity task as compared to using networks that were initialized with ImageNet pre-trained weights (The input domain of ImageNet is significantly different than the WZIS dataset). We still use the splits defined in Section 4.2.1, only using the labels to create appropriate triplets.

#### 4.4.2. Results

We report the results in terms of mean Average Precision (mAP), which is a well established metric in the context of similarity matching. The mAP should be interpreted as—higher the better, with an optimal score of 1.0. Table 3 clearly demonstrates that the Classification and Tagging pre-trained networks outperform the ImageNet baseline networks by a significant margin on all subsets of the dataset (as defined in Section 4.2.1). As with the other two tasks, we can see here that the DenseNet outperforms the ResNet.

## 5. Comparison of Different Approaches

In the previous sections, we evaluated our different approaches on test sets that are appropriate to the individual approaches. However, in order to evaluate the effectiveness of the various systems for the use of researchers in the humanities, we need a different evaluation approach. Therefore, we apply the various trained systems from Section 4 to the task of query-based image retrieval on several expert-defined test sets (see Section 3.3) that are not part of the splits used in any of the experiments from Section 4.

For every query in TestSet50, we use the different systems to retrieve an ordered list of results. The results of the systems are then compared against the expert-defined results using the mean Average Precision (mAP) metric to determine the best performing system. The best performing system is further evaluated on the test sets using the Normalized Discounted Cumulative Gain (NDCG) [63,64] metric in addition to the mAP metric. The NDCG metric can be intuitively understood as measuring the quality of the returned results in a more fine-grained way compared to the mAP. This is done by assigning multiple possible relevance levels to each response of a given query. This means that for a given query and a collection of documents, a system that returns relevant results at a higher rank will have a higher NDCG score compared to a system that returns the same results but at a lower rank.

In Table 4, we see the mAP scores for the various systems on TestSet 50. While all the systems achieve over 0.9 mAP, we can clearly see that the DenseNet-121 is better than the ResNet-18 consistently across all the approaches. However, we see that networks trained exclusively for classification/tagging and then fine-tuned for similarity matching perform the best, followed by networks trained only for similarity matching. Networks that were trained for only classification/tagging perform the worst.

**Figure 5:**
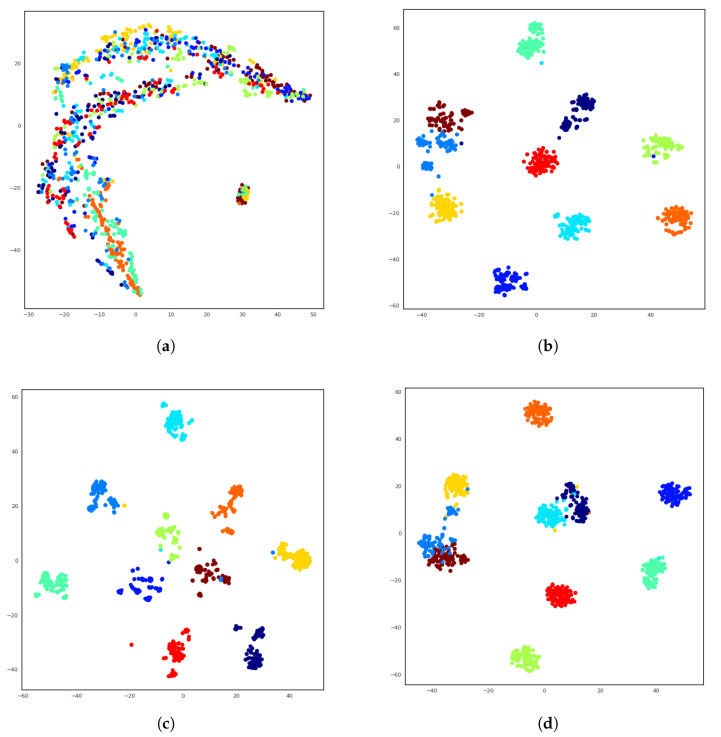
t-Distributed Stochastic Neighbor Embedding (t-SNE) [65] visualization of the TestSet1000 in the latent embedding space. Different colors denotes samples belonging to different classes. (**a**) Randomly initialized; (**b**) Trained for classification; (**c**) Trained for multi-label; (**d**) Trained for similarity.

Figure 5 shows the t-Distributed Stochastic Neighbor Embedding (t-SNE) [65] visualization of the embeddings for TestSet1000. It is interesting to note here that both the classification and tagging networks produces multiple sub-clusters per class. We speculate that these sub-clusters could correspond to the same watermark depicted in different ways. Rauber et al. [66] observed a similar phenomenon when analyzing networks trained on the Street View House Number Dataset, where the a cluster of the same digit had sub-clusters based on the color of the image (light text on dark background or vice-versa).

Finally, we evaluate the best performing network (tagging fine-tuned for similarity) on TestSet50 and compute the NDCG (higher the better, with optimal score of 1.0), which takes into account the relevance and the position of each result [63]. The result in Table 5 shows that our approach works perfectly for assigning the correct class on a binary level relevant/irrelevant (represented by the mAP metric), but the precision of the ranking (represented by the NDCG metrics) can still be improved.

## 6. Comparison with State-of-the-Art

In this section, we compare the performance of our best system against the LIRE and IOSB systems (see Section 2.2.1 and Section 2.2.2). The evaluation metrics used here include the Precision@35, mAP (see Section 4.4.2) and the average rank of the first relevant match and the best possible match. Precision@k is the ratio of the top-k documents that are relevant to the query.

**Figure 6:**
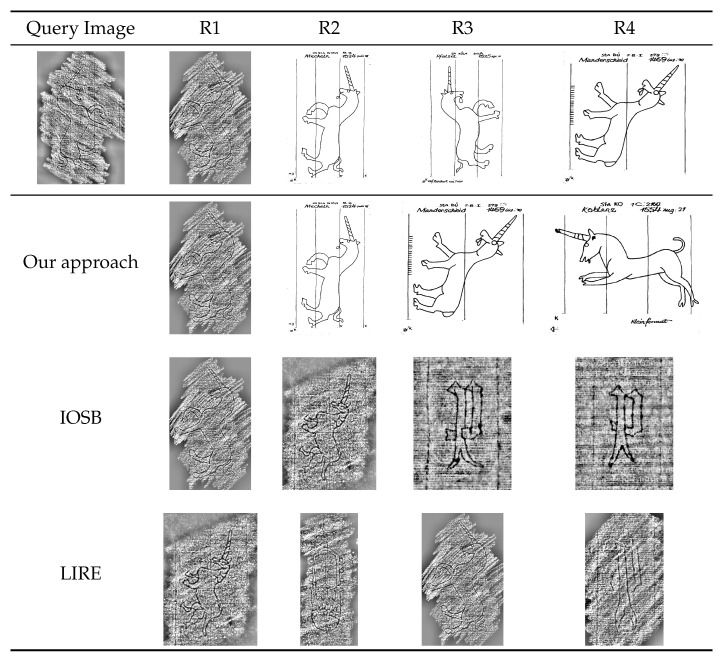
The top row shows a query image belonging to class Fabulous Creature/Unicorn and the first 4 results annotated by an expert (used as ground truth for evaluation). The second row shows the results (R1 through R4) retrieved by our approach when pre-trained for tagging and fine-tuned for similarity. Notice that the images all belong to the correct class. The third row shows the results retrieved by the IOSB system [67] and the last row those of the LIRE system [68]. In these cases, occasionally some of the retrieved images belong to another class. It is also clear that the IOSB and LIRE systems are much less robust against different reproduction techniques, since these systems tend to return similar reproduction techniques (rubbings, radiographies) rather than similar motifs.

From Table 6 and Table 7 we can see that our best performing system outperforms both the other systems by a significant margin on both TestSet50 and TestSet1000. We achieve a 1.0 mAP score on TestSet50, which implies that our system retrieves the correct class for every query in the test set. This can also be seen in Figure 6, where our system retrieves the correct results for the query image (although in a different order).

This is significant as the top results contain images that are represented in different manners. Our system is able to handle this cross-depiction, while the other systems tend to retrieve images that are depicted in the same manner as the query image. In Table 8, we see that for a query image, our system on average returns a relevant image at the first location, and the most appropriate result for the query image is found around the second or third location.

## 7. Conclusions and Future Directions

In this paper, we showed that deep learning-based approaches can be very useful for classifying and analyzing watermarks in historical documents, despite the fact that the watermarks are depicted in various manners. Thereby we found evidence supporting two hypotheses:

1. using deep learning for similarity matching is efficient and overcomes the problems of the standard human-made classification system used for structuring watermarks.
2. the high-level representations learned by deep learning models are useful for classification and similarity matching of the motifs, despite the fact that they are depicted in different ways in the original images.

In various experiments, we assessed the performance of two state-of-the-art CNN architectures in three different experimental settings (classification, tagging and similarity matching) on a large dataset of cross-depicted watermarks. These deep-learning-based approaches are better than available similarity-matching tools and reach a perfect retrieval rate of relevant motifs. Such a system can be very helpful for experts when classifying watermarks and cataloging the corresponding manuscripts as similar watermarks are found based on their visual appearance and not based on a hierarchical and inflexible classification system.

Our work (Preliminary work in our paper [12], followed by an in-depth study in this paper) is a pioneering step in evaluating deep-learning-based methods on a large dataset of cross-depicted motifs. It indicates that a promising future direction is the development of an image-based similarity matching tool which is not only useful for watermarks but could be useful in other settings, such as retrieval of trademark images [69]. Contrary to indications from previous work regarding the limited effectiveness of deep learning when dealing with cross-depicted data, our results indicate that deep learning is effective at dealing with cross-depicted watermarks. This could be in part due to limited variability of the cross-depictions within the watermarks when compared to cross-depiction in other domains (e.g., between a photograph and artwork of the same object). An analysis of why deep learning works in this setting along with a deeper analysis of the classification and multi-labelling results would be a promising future direction, i.e., it would be interesting to see what an optimal classification rate is (Please note that the classification system is “noisy”, as discussed in Section 3.2.1 and therefore a 100% accuracy would be an ill-posed problem).

## Figures and Tables

**Table 1:** Macro-averaged F1-scores for both architectures on the Classification task. The networks were initialized using weights obtained after training on the ImageNet dataset.

	Training Set	Validation Set	Test Set
ResNet18	99.12%	84.51%	85.46%
DenseNet121	99.33%	88.27%	88.3%

**Table 2:** Multi-label tagging performance (mean IoU).

	Training Set	Validation Set	Test Set
ResNet18	91.14%	74.54%	74.86%
DenseNet121	93.30%	79.43%	79.57%

**Table 3:** Similarity matching performance (mAP).

	ResNet18	DenseNet121
Baseline	0.885	0.928
Classification Pre-trained	0.929	0.951
Tagging Pre-trained	0.923	**0.952**

**Table 4:** A comparison of the performance of various models measured on the queries of TestSet50. All models were used in a similarity matching setting, with Classification and Tagging models used either with (fine-tuned) or without further training (baseline). For both Classification and Tagging tasks, the models were modified by removal of the final layer in order to use the activations of the penultimate layer as an embedding in a high-dimensional space.

Metric: mAP		ResNet-18	DenseNet-121
Classification	baseline	0.939	0.964
	fine-tuned	0.985	0.997
Tagging	baseline	0.91	0.921
	fine-tuned	0.953	**1.0**
Similarity	*n/a*	0.966	0.999

**Table 5:** mAP and NDCG scores of the best performing system on TestSet50 broken down by category.

	Bull’s Head	Letter P	Crown	Unicorn	Grape	Average
mAP	1.0	1.0	1.0	1.0	1.0	1.0
NDCG	0.96	0.80	0.83	0.93	0.93	0.89

**Table 6:** Performances of the three systems on TestSet1000.

	IOSB	LIRE	Proposed
Precision@35	60%	20%	**91.4%**

**Table 7:** mAP scores of the three systems on TestSet50.

	Bull’s head	Letter P	Crown	Unicorn	Grape	Average
IOSB	0.45	0.24	0.42	0.34	0.75	0.44
LIRE	0.55	0.24	0.48	0.14	0.48	0.38
Proposed	**1**	**1**	**1**	**1**	**1**	**1**

**Table 8:** Average rank of the first relevant match measured on TestSet50. The metric is interpreted as the lower the better with lower bound 1.

	IOSB	LIRE	Our
First relevant match	1.78	1.82	**1**
Best match	7.75	10.6	**2.44**

## References

[1] Rückert P. (2006). Ochsenkopf und Meerjungfrau. Wasserzeichen des Mittelalters.

[2] Haidinger A. (2004). Datieren mittelalterlicher Handschriften mittels ihrer Wasserzeichen. Anz. Phil. Hist. Kl. ÖSterreichischen Akad. Wiss..

[3] Bange E., Meyer C., Schultz S., Schneidmüller B. (2015). Wasserzeichen als Quelle zur Wirtschafts- und Sozialgeschichte: Eine Studie am Beispiel der Luxemburger Kontenbücher. Papier im mittelalterlichen Europa. Herstellung und Gebrauch.

[4] Frauenknecht E., Rückert P., Frauenknecht E. (2015). Symbolik im Papier? Die Tiara als Wasserzeichen in der Kanzlei Kaiser Friedrichs III. Wasserzeichen und Filigranologie. Beiträge einer Tagung zum 100. Geburtstag von Gerhard Piccard (1909–1989).

[5] Stutzmann D., Brookes S., Rehbein M., Stokes P. (2018). Variability as a Key Factor For Understanding Medieval Scripts: The ORIFLAMMS Project (ANR-12-CORP-0010).

[6] Kestemont M., Christlein V., Stutzmann D. (2017). Artificial Paleography: Computational Approaches to Identifying Script Types in Medieval Manuscripts. Speculum.

[7] Bell P., Schlecht J., Ommer B. (2013). Nonverbal communication in medieval illustrations revisited by computer vision and art history. Vis. Resour..

[8] Yarlagadda P., Monroy A., Carqué B., Ommer B. (2013). Towards a computer-based understanding of medieval images. Scientific Computing and Cultural Heritage.

[9] Hall P., Cai H., Wu Q., Corradi T. (2015). Cross-depiction problem: Recognition and synthesis of photographs and artwork. Comput. Vis. Med..

[10] Smeulders A.W., Worring M., Santini S., Gupta A., Jain R. (2000). Content-based image retrieval at the end of the early years. IEEE Trans. Pattern Anal. Mach. Intell..

[11] Yoon J. (2015). Image Retrieval Practice and Research. Encyclopedia of Information Science and Technology.

[12] Pondenkandath V., Alberti M., Eichenberger N., Ingold R., Liwicki M. (2018). Identifying cross-depicted historical motifs. Proceedings of the 2018 16th International Conference on Frontiers in Handwriting Recognition (ICFHR).

[13] Szegedy C., Zaremba W., Sutskever I., Bruna J., Erhan D., Goodfellow I., Fergus R. Intriguing properties of neural networks. Proceedings of the International Conference on Learning Representations.

[14] Karpathy A. What I Learned from Competing Against a ConvNet on ImageNet. http://karpathy.github.io/2014/09/02/what-i-learned-from-competing-against-a-convnet-on-imagenet/.

[15] Levandowsky M., Winter D. (1971). Distance between Sets.

[16] Shen X., Efros A.A., Aubry M. Discovering visual patterns in art collections with spatially-consistent feature learning. Proceedings of the IEEE Conference on Computer Vision and Pattern Recognition.

[17] Huang F., Jin C., Zhang Y., Weng K., Zhang T., Fan W. (2018). Sketch-based image retrieval with deep visual semantic descriptor. Pattern Recognit..

[18] Crowley E., Zisserman A. (2014). The State of the Art: Object Retrieval in Paintings using Discriminative Regions. Proceedings of the British Machine Vision Conference.

[19] Hu R., Collomosse J. (2013). A performance evaluation of gradient field HOG descriptor for sketch based image retrieval. Comput. Vis. Image Underst..

[20] Rakthanmanon T., Zhu Q., Keogh E.J. (2012). Efficiently Finding Near Duplicate Figures in Archives of Historical Documents. J. Multimed..

[21] En S., Petitjean C., Nicolas S., Heutte L. (2016). A scalable pattern spotting system for historical documents. Pattern Recognit..

[22] Ginosar S., Haas D., Brown T., Malik J. (2014). Detecting people in cubist art. Proceedings of the European Conference on Computer Vision.

[23] Rauber C., Tschudin P., Startchik S., Pun T. (1996). Archival and retrieval of historical watermark images. Proceedings of the 3rd IEEE International Conference on Image Processing.

[24] Belov V.V., Esipova V.A., Kalaida V.T., Klimkin V.M. (1999). Physical and Mathematical Methods for the Visualisation and Identification of Watermarks. Solanus.

[25] Brown A.J.E., Mulholland R., Graham M., Riley J., Vassilev V., Eakins J., Furness K. (2002). When Images Work Faster than Words The Integration of Content-Based Image Retrieval with the Northumbria Watermark Archive. Restaurator.

[26] Riley K.J., Edwards J.D., Eakins J.P. (2003). Content-Based Retrieval of Historical Watermark Images: II—Electron Radiographs.

[27] Brunner G. (2006). Structure Features for Content-Based Image Retrieval And Classification Problems. Ph.D. Thesis.

[28] Otal H.M., Lubbe J.C.A.V.D. (2008). Isolation and Identification of Identical Watermarks within Large Databases. Elektronische Medien & Kunst, Kultur, Historie Konferenzband EVA (2008 Berlin); 12–14 November 2008 in Den Staatlichen Museen Zu Berlin Am Kulturforum Potsdamer Platz; Die 15. Berliner Veranstaltung der Internationalen EVA-Serie Electronic Imaging & the Visual Arts.

[29] Picard D., Henn T., Dietz G. (2016). Non-negative dictionary learning for paper watermark similarity. Proceedings of the 2016 50th Asilomar Conference on Signals, Systems and Computers.

[30] Shen X., Pastrolin I., Bounou O., Gidaris S., Smith M., Poncet O., Aubry M. (2019). Large-Scale Historical Watermark Recognition: Dataset and a new consistency-based approach. arXiv.

[31] Veltkamp R.C., Tanase M. (2000). Content-Based Image Retrieval Systems: A Survey.

[32] Kosch H., Maier P. (2010). Content-Based Image Retrieval Systems-Reviewing and Benchmarking. J. Digit. Inf. Manag..

[33] Crabbe S., Ambs P., Black S., Wilkinson C., Bikker J., Herz N., Manger D., Pape R., Seibert H. (2013). Results of the FASTID Project.

[34] Marques O., Lux M. (2012). Visual information retrieval using Java and LIRE. Proceedings of the 35th International ACM SIGIR Conference on Research and Development in Information Retrieval.

[35] Deng J., Dong W., Socher R., Li L.J., Li K., Li F.-F. ImageNet: A large-scale hierarchical image database. Proceedings of the 2009 IEEE Conference on Computer Vision and Pattern Recognition.

[36] Krizhevsky A., Sutskever I., Geoffrey E.H. (2012). ImageNet Classification with Deep Convolutional Neural Networks. Advances in Neural Information Processing Systems 25 (NIPS2012).

[37] He K., Zhang X., Ren S., Sun J. Delving Deep into Rectifiers: Surpassing Human-Level Performance on ImageNet Classification. Proceedings of the IEEE International Conference on Computer Vision (ICCV).

[38] Hu J., Shen L., Sun G. Squeeze-and-Excitation Networks. Proceedings of the IEEE Conference on Computer Vision and Pattern Recognition (CVPR).

[39] Ittelson W.H., Kilpatrick F.P. (1951). Experiments in perception. Sci. Am..

[40] Alberti M., Pondenkandath V., Würsch M., Bouillon M., Seuret M., Ingold R., Liwicki M. Are You Tampering With My Data?. Proceedings of the European Conference on Computer Vision (ECCV).

[41] Piccard G. (1961–1997). Die Wasserzeichenkartei Piccard im Hauptstaatsarchiv Stuttgart, 17 Findbücher in 25 Bänden.

[42] Briquet C.M. (1907). Les Filigranes: Dictionnaire Historique des Marques du Papier dès leur Apparition vers 1282 Jusqu’en 1600.

[43] Wenger E., Eckhardt W., Neumann J., Schwinger T., Staub A. (2016). Metasuche in Wasserzeichendatenbanken (Bernstein-Projekt): Herausforderungen für die Zusammenführung heterogener Wasserzeichen-Metadaten. Wasserzeichen—Schreiber—Provenienzen: Neue Methoden der Erforschung und Erschließung von Kulturgut im Digitalen Zeitalter: Zwischen Wissenschaftlicher Spezialdisziplin und Catalog Enrichment.

[44] Palmer N.F., Rückert P., Maier G. (2007). Verbalizing Watermarks: The Question of a Multilingual Database. Piccard-Online. Digitale Präsentationen von Wasserzeichen und ihre Nutzung.

[45] Frauenknecht E., Meyer C., Schultz S., Schneidmüller B. (2015). Papiermühlen in Württemberg. Forschungsansätze am Beispiel der Papiermühlen in Urach und Söflingen. Papier im mittelalterlichen Europa. Herstellung und Gebrauch.

[46] Wolf C., Rehbein M., Sahle P., Schaßan T. (2009). Aufbau eines Informationssystems für Wasserzeichen in den DFG-Handschriftenzentren. Kodikologie und Paläographie im Digitalen Zeitalter.

[47] Karnaukhov V., Vahtikari V., Hakkarainen M., Nurminen A. (2011). Methods and Tools for Watermark Digital Processing, Archiving and Dating. EIKONOPOIIA. Digital Imaging of Ancient Textual Heritage.

[48] Neuheuser H.P., Märgner V., Meinlschmidt P. (2005). Wasserzeichendarstellung mit Hilfe der Thermographie. ABI Tech..

[49] Eckhardt W. (2014). Erschließung und Bildliche Dokumentation von Wasserzeichen in Online-Datenbanken.

[50] Frauenknecht E., Eckhardt W., Neumann J., Schwinger T., Staub A. (2016). Von Wappen und Ochsenköpfen: Zum Umgang mit großen Motivgruppen im ‘Wasserzeichen-Informationssystem’ (WZIS). Wasserzeichen—Schreiber—Provenienzen: Neue Methoden der Erforschung und Erschließung von Kulturgut im digitalen Zeitalter: Zwischen Wissenschaftlicher Spezialdisziplin und Catalog Enrichment.

[51] Alberti M., Pondenkandath V., Würsch M., Ingold R., Liwicki M. DeepDIVA: A Highly-Functional Python Framework for Reproducible Experiments. Proceedings of the 2018 16th International Conference on Frontiers in Handwriting Recognition (ICFHR).

[52] Hochreiter S., Schmidhuber J. (1997). Long Short-Term Memory. Neural Comput..

[53] He K., Zhang X., Ren S., Sun J. Deep residual learning for image recognition. Proceedings of the IEEE Conference on Computer Vision and Pattern Recognition.

[54] Huang G., Liu Z., Van Der Maaten L., Weinberger K.Q. Densely Connected Convolutional Networks. Proceedings of the IEEE Conference on Computer Vision and Pattern Recognition.

[55] LeCun Y., Bottou L., Bengio Y., Haffner P. (1998). Gradient-based learning applied to document recognition. Proc. IEEE.

[56] Russakovsky O., Deng J., Su H., Krause J., Satheesh S., Ma S., Huang Z., Karpathy A., Khosla A., Bernstein M. (2015). ImageNet Large Scale Visual Recognition Challenge. Int. J. Comput. Vis. IJCV.

[57] Studer L., Alberti M., Pondenkandath V., Goktepe P., Kolonko T., Fischer A., Liwicki M., Ingold R. (2019). A Comprehensive Study of ImageNet Pre-Training for Historical Document Image Analysis. Proceedings of the 2019 15th International Conference on Document Analysis and Recognition (ICDAR).

[58] Singh M.S., Pondenkandath V., Zhou B., Lukowicz P., Liwicki M. (2017). Transforming Sensor Data to the Image Domain for Deep Learning—An Application to Footstep Detection. Proceedings of the 2017 International Joint Conference on Neural Networks (IJCNN).

[59] Zagoruyko S., Komodakis N. Learning to compare image patches via convolutional neural networks. Proceedings of the IEEE Computer Society Conference on Computer Vision and Pattern Recognition.

[60] Han X., Leung T., Jia Y., Sukthankar R., Berg A.C. MatchNet: Unifying feature and metric learning for patch-based matching. Proceedings of the IEEE Computer Society Conference on Computer Vision and Pattern Recognition.

[61] Hoffer E., Ailon N. (2015). Deep metric learning using triplet network. Lecture Notes in Computer Science (Including Subseries Lecture Notes in Artificial Intelligence and Lecture Notes in Bioinformatics).

[62] Balntas V., Riba E., Ponsa D., Mikolajczyk K. (2016). Learning Local Feature Descriptors with Triplets and Shallow Convolutional Neural Networks.

[63] Yao T., Wang G., Yan L., Kong X., Su Q., Zhang C., Tian Q. (2019). Online latent semantic hashing for cross-media retrieval. Pattern Recognit..

[64] Christopher D.M., Prabhakar R., Hinrich S. (2008). Introduction to Information Retrieval. An Introd. Inf. Retr..

[65] Maaten L.v.d., Hinton G. (2008). Visualizing data using t-SNE. J. Mach. Learn. Res..

[66] Rauber P.E., Fadel S.G., Falcao A.X., Telea A.C. (2017). Visualizing the Hidden Activity of Artificial Neural Networks. IEEE Trans. Vis. Comput. Graph..

[67] Manger D. Large-scale tattoo image retrieval. Proceedings of the 2012 9th Conference on Computer and Robot Vision (CRV 2012).

[68] Lux M. Content based image retrieval with LIRE. Proceedings of the 19th ACM International Conference on Multimedia—MM ’11.

[69] Ravela S., Manmatha R., Croft W.B. (2005). Retrieval of Trademark and Gray-Scale Images Using Global Similarity.

